# High‐Quality Graphene Using Boudouard Reaction

**DOI:** 10.1002/advs.202200217

**Published:** 2022-02-20

**Authors:** Artem K. Grebenko, Dmitry V. Krasnikov, Anton V. Bubis, Vasily S. Stolyarov, Denis V. Vyalikh, Anna A. Makarova, Alexander Fedorov, Aisuluu Aitkulova, Alena A. Alekseeva, Evgeniia Gilshtein, Zakhar Bedran, Alexander N. Shmakov, Liudmila Alyabyeva, Rais N. Mozhchil, Andrey M. Ionov, Boris P. Gorshunov, Kari Laasonen, Vitaly Podzorov, Albert G. Nasibulin

**Affiliations:** ^1^ Skolkovo Institute of Science and Technology Nobel str. 3 Moscow 121205 Russia; ^2^ Moscow Institute of Physics and Technology Institute Lane 9 Dolgoprudny Russia; ^3^ Insitute of Solid State Physics (RAS) Academician Ossupyan str. 2 Chernogolovka Russia; ^4^ Dukhov Research Institute of Automatics (VNIIA) Moscow 127055 Russia; ^5^ HSE University Myasnitskaya 20 Moscow 101000 Russia; ^6^ National University of Science and Technology MISIS Moscow 119049 Russia; ^7^ Donostia International Physics Center (DIPC) Donostia‐San Sebastián 20018 Spain; ^8^ IKERBASQUE Basque Foundation for Science Bilbao 48011 Spain; ^9^ Physikalische Chemie Institut für Chemie und Biochemie Freie Universität Berlin Arnimallee 22 Berlin 14195 Germany; ^10^ IFW Dresden POB 270116 Dresden D‐01171 Germany; ^11^ Empa Swiss Federal Laboratories for Materials Science and Technology Ueberlandstrasse 129 Duebendorf 8600 Switzerland; ^12^ Boreskov Institute of Catalysis SB RAS Novosibirsk 630090 Russia; ^13^ National Research Nuclear University MEPhI (Moscow Engineering Physics Institute) Moscow 115409 Russia; ^14^ Aalto University P.O. Box 16100 Aalto FI‐00076 Finland; ^15^ Department of Physics Rutgers University Piscataway NJ 08854 USA

**Keywords:** Boudouard reaction, carbon monoxide, copper, chemical vapor deposition, graphene

## Abstract

Following the game‐changing high‐pressure CO (HiPco) process that established the first facile route toward large‐scale production of single‐walled carbon nanotubes, CO synthesis of cm‐sized graphene crystals of ultra‐high purity grown during tens of minutes is proposed. The Boudouard reaction serves for the first time to produce individual monolayer structures on the surface of a metal catalyst, thereby providing a chemical vapor deposition technique free from molecular and atomic hydrogen as well as vacuum conditions. This approach facilitates inhibition of the graphene nucleation from the CO/CO_2_ mixture and maintains a high growth rate of graphene seeds reaching large‐scale monocrystals. Unique features of the Boudouard reaction coupled with CO‐driven catalyst engineering ensure not only suppression of the second layer growth but also provide a simple and reliable technique for surface cleaning. Aside from being a novel carbon source, carbon monoxide ensures peculiar modification of catalyst and in general opens avenues for breakthrough graphene‐catalyst composite production.

## Graphical abstract

Unique features of Boudouard reaction facilitate high‐purity, wafer‐scale single‐crystal graphene on metal catalysts.

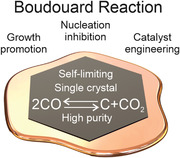

## 1 Introduction

Graphene has become a milestone discovery in contemporary solid‐state physics and materials science.^[^ ^1^ ^]^ Properties of novel 2D systems based on graphene have opened the avenue for high‐performance devices^[^ ^2^, ^3^ ^]^ and novel physical phenomena.^[^ ^4^, ^5^ ^]^ Nevertheless, the method for the state‐of‐the‐art graphene, i.e., “natural” exfoliation,^[^ ^1^, ^2^, ^3^ ^]^ is strictly limited in terms of the sample size and geometry. Therefore, a large‐scale graphene synthesis is a challenge to bring lab‐scale prototypes to a wide range of applications. One of the most advanced approaches to graphene preparation is chemical vapor deposition (CVD).^[^ ^6^, ^7^ ^]^ This method comprises catalytic decomposition of gaseous carbon sources (typically hydrocarbons) on metallic substrates. The CVD synthesis of single‐crystal monolayer graphene is an unprecedented challenge for catalysis: the cycle of carbon precipitation should be terminated after the first graphitic layer, and carbon‐, hydrogen‐, and oxygen‐based intermediates should control the nucleation of new seeds.

During the past decade, wafer‐scale single‐crystal graphene synthesis was achieved using hydrocarbons such as CH_4_^[^ ^8^, ^9^, ^10^ ^]^ and C_2_H_4_^[^ ^11^, ^12^ ^]^ on the surface of metallic (Cu, Ni/Cu, Ru)^[^ ^8^, ^9^, ^11^, ^12^ ^]^ and semiconducting (Ge)^[^ ^10^ ^]^ substrates. One of the strategies to obtain single‐crystal graphene is the alignment of the graphene nuclei that prevents defect formations caused by grain boundaries.^[^ ^8^, ^10^, ^11^ ^]^ This method demands a single‐crystalline metallic foil, which requires additional technological steps. Another strategy relies on the inhibition of graphene nucleus density so that each seed grows up into a large graphene crystal.^[^ ^7^, ^9^, ^13^ ^]^ Such an approach requires a balance between nucleation control and the growth rate of a single grain. In this case, the pressure within the reactor plays a dominant role to control the nucleation process.^[^ ^13^ ^]^ Remarkable progress in obtaining large graphene samples with outstanding electronic properties and superior crystallinity was achieved using molten and resolidified substrates.^[^ ^14^, ^15^ ^]^ However, according to the recent review,^[^ ^7^ ^]^ rapid synthesis technique facilitating single grain growth is still a challenge.

A carbon source with specific stability to prevent nucleation of multiple grains by both pyrolysis and surface decomposition may help to overcome the abovementioned problems. Surprisingly, one of the simplest carbon sources, carbon monoxide, has acquired little attention. Though the first multilayer graphitic structures were reported as early as in the 1950s^[^ ^16^, ^17^ ^]^ and “graphene‐like structures”^[^ ^18^, ^19^ ^]^ were recently observed, CO has never been used for the CVD synthesis of monolayer graphene on a metallic substrate. Carbon monoxide is truly a unique carbon source. Unlike the conventional methods based on pyrolysis of hydrocarbons requiring hydrogen to control carbon intermediates, the unique thermodynamics of the Boudouard reaction, 2CO ⇆ C + CO_2_, under high temperatures favors CO, not the carbon precipitation.^[^ ^20^, ^21^ ^]^ The high stability of CO molecule restricts noncatalytic pyrolysis,^[^ ^22^ ^]^ and, thereby, formation of amorphous deposits or second graphitic layer. Moreover, carbon monoxide appears to have a peculiar interaction with the surface of transition metals,^[^ ^23^ ^]^ especially with copper.^[^ ^24^ ^]^

Taking into account all benefits of CO utilization, the absence of the method for monolayer graphene synthesis is even more interesting, since the well‐studied Boudouard reaction has been actively used for the synthesis of graphite^[^ ^25^, ^26^ ^]^ and single‐walled carbon nanotubes (SWCNTs)^[^ ^27^, ^28^, ^29^ ^]^ both on lab‐ and industrial scales. Indeed, HiPco^[^ ^30^ ^]^ (high‐pressure CO) was one of the very first technologies to deliver a high yield SWCNT synthesis, while the combination of the aerosol CVD method with CO provides state‐of‐the‐art transparent conducting films.^[^ ^31^ ^]^ It should be mentioned that SWCNT growth based on the Boudouard reaction allows nucleation control by the addition of CO_2_.^[^ ^28^, ^29^, ^32^ ^]^ At the same time, CO_2_ is also a superior pre‐treatment “cleaning” tool during the graphene synthesis.^[^ ^33^ ^]^ However, the utilization of CO_2_ with hydrocarbons to control nucleation and growth processes during graphene synthesis is limited.^[^ ^34^ ^]^ In contrast, the Boudouard reaction allows the implementation of both nucleation control and perfect cleaning by CO_2_. Finally, the CO‐based synthesis route is completely hydrogen‐free, favorable in atmospheric or high‐pressure CVD implementations.^[^ ^28^, ^29^ ^]^

## 2 Results

**Figure 1:**
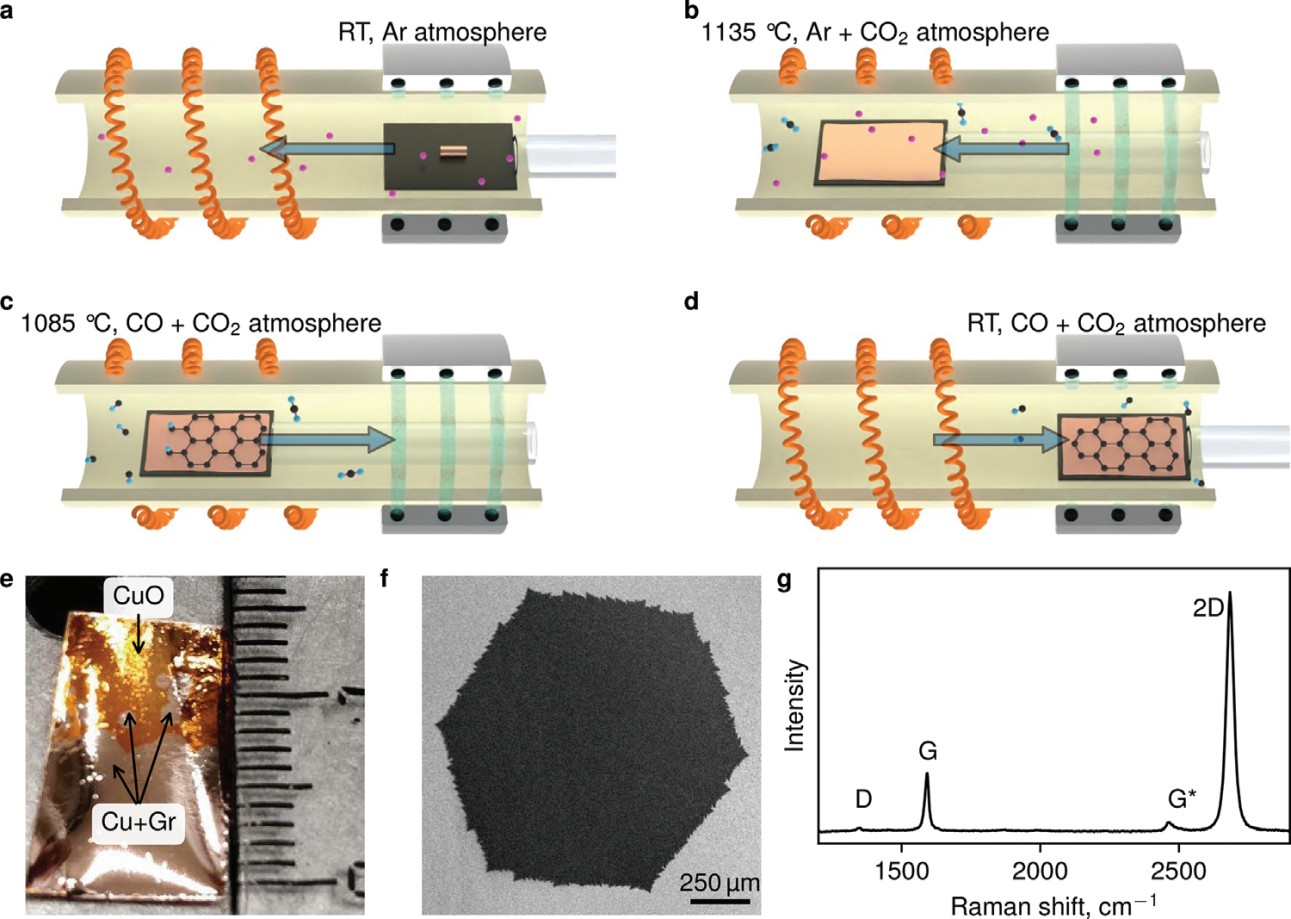
Chemical vapor deposition (CVD) synthesis of graphene from carbon monoxide. a) A molybdenum wetting‐layer foil with a polycrystalline copper chunk/foil is purged with argon gas. b) The substrate is loaded into the hot zone (1135 °C) of the furnace for melting and cleaning in the Ar/CO_2_ atmosphere. c) The sample is cooled down in the CO + CO_2_ atmosphere to 1085 °C for graphene growth and copper solidification. d) The sample is rapidly transferred into the water‐cooled flange region. e) Optical photograph of the polycrystalline sample, and f) scanning electron microscopy (SEM) image of an individual single‐crystal graphene grain on the surface of the copper catalyst, respectively. g) A typical Raman spectrum of graphene transferred from a copper to a quartz substrate.

Here, we propose an elegant solution to the challenge of single‐crystal graphene synthesis based on a “pyrolysis‐free” process of carbon monoxide catalytic decomposition on a copper heated to the temperature slightly above its melting point (1083–1085 °C). We have constructed a horizontal tubular hot‐wall reactor equipped with a fast load‐in∖out ceramic manipulator (**Figure** **1**a–d). After purging a polycrystalline chunk of Cu catalyst placed on top of Mo‐foil wetting layer with an ultra‐high purity argon gas (Figure 1a) and melting it in a CO_2_ rich atmosphere at 1135 °C (Figure 1b), a CO/CO_2_ gaseous mixture is introduced into the reactor chamber, while the temperature is decreased to 1085 °C (Figure 1c). After a certain time, when the synthesis is complete, the sample is rapidly moved to the water‐cooled zone (Figure 1d). The system operates at nearly atmospheric pressure with a possibility to increase it up to 4 bar and higher. To visualize graphene grains, we heat treat the sample at ≈120 °C on a hot plate in air boosting oxidation of the exposed copper surface, but not the area protected from oxidation by graphene, leading to the appearance of a contrast as shown in Figure 1e (for more details, see Supporting Information).

**Figure 2:**
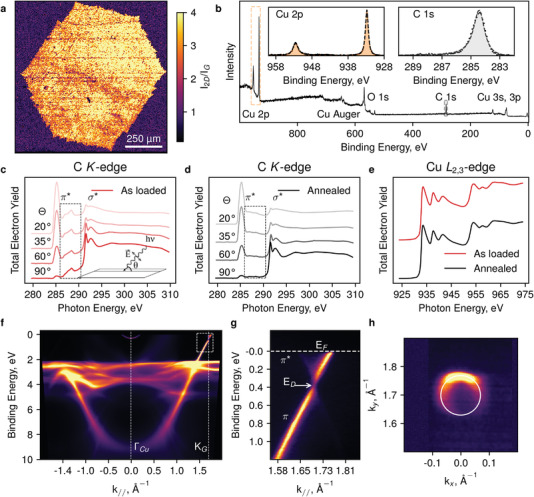
Spectroscopy of graphene produced by Boudouard reaction. a) Raman spectroscopy map of *I*_2D_/*I*_G_ ratio of a mm‐size individual single‐crystal graphene grain transferred to a SiO_2_ substrate. b) A survey XPS spectrum of the sample with insets showing XPS core‐level spectra of C 1s and Cu 2p lines. c,d) C *K*‐edge NEXAFS spectra as a function of *θ* (see inset in panel c) for as‐loaded and annealed (≈300 °C) samples of graphene on copper. Inset in panel c shows the geometry of the experiment. e) Cu *L*_2,3_‐edge NEXAFS spectra of the same sample before and after annealing. These results demonstrate the ultimate cleanliness of the Cu‐Gr interface. f) Photoemission spectroscopy map in M‐Γ‐K direction of the same polycrystalline Gr‐Cu sample, with the Shockley surface state of Cu clearly observed near the Γ point and close to *E*_F_. g) A zoom of the graphene spectrum around the K point. h) Fermi surface of graphene.

**Figure 3:**
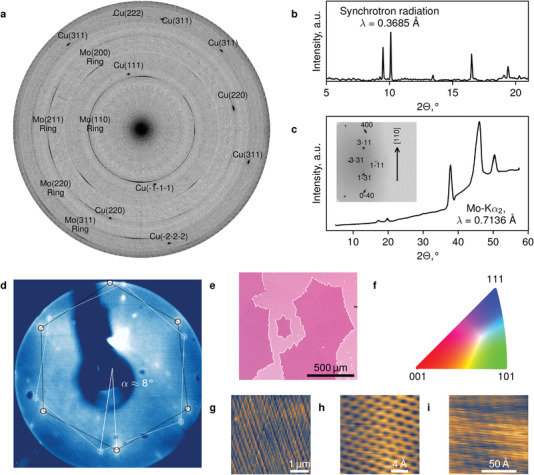
The structure of the graphene‐copper system. a) Raw and b) averaged over azimuthal angle plots of XRD data collected in the transmission geometry using synchrotron radiation. c) Averaged over azimuthal angle roentgenogram (raw data shown in the inset) in the reflection geometry indicating single‐crystalline copper under graphene layer. d)LEED data (*E*_e_ = 48 eV) from an individual grain of ≈4 mm in size. e) EBSD map in the form of inverse pole figures for sample's normal direction. Color coding legend is indicated in the panel (f). g) AFM topography image of the nanostructured copper underneath graphene. h,i) STM images of graphene lattice and Moire pattern, respectively.

Catalytic disproportionation of carbon monoxide leads to the formation of graphene grains of up to several mm in size (Figure 1e,f). These are purely monolayer structures (as evidenced by Raman spectroscopy mapping: the typical individual spectrum and a map are shown in Figure 1g and **Figure** **2**a, respectively), providing the distribution of *I*_2D_/*I*_G_ greater than 2 over the entire area of the sample. Dendritic boundaries are likely formed due to high growth rates,^[^ ^13^ ^]^ while the produced domains still preserve hexagonal shape that suggests a single‐crystal nature of the layer. X‐ray photoelectron spectroscopy (XPS) data collected from the graphene‐coated copper surface reveal the outstanding purity of both the graphene and the copper substrate (Figure 2b). Obtained structures had extremely low sticking coefficients as after several weeks of exposure to air before the introduction of samples to electron spectrometer excellent low energy electron diffraction (LEED) patterns (**Figure** **3**d) and XPS (Figure 2b) spectra were observed. The position of C 1s line (right inset in Figure 2b) at 284.4 eV and its form are attributed to pure sp^2^ carbon, described in the literature as a “free‐standing” non‐interacting graphene,^[^ ^32^ ^]^ which is not contaminated and has no additional bonds often observed for the hydrocarbon derived samples.^[^ ^35^, ^36^, ^37^, ^38^ ^]^ At the same time, the state of the catalyst appears to correspond to a pure unoxidized copper, Cu^0^,^[^ ^39^ ^]^ as supported by Cu 2p (932.7 eV) in Figure 2b (the left inset) and Auger Cu_LMM_ lines (*E*_K_ = 918.7 eV, see Figure S1, Supporting Information). Minor contaminations revealed by weak O 1s (core level in Figure S1, Supporting Information) line in the XPS spectrum (obtained without vacuum sample annealing) were analyzed by the near edge X‐ray absorption fine structure (NEXAFS) spectroscopy.^[^ ^40^ ^]^ These trace contaminants of various carbon bonds (regions marked by dashed rectangles in Figure 2c,d) can be removed by a 300 °C annealing in ultra‐high vacuum conditions. Remarkably, Cu *L*_2,3_‐edge signal (Figure 2e), indicative of pure metallic copper^[^ ^41^ ^]^ only weakly interacting with graphene, does not change during annealing. It is indicative of the atmospheric origin of the oxygen line in the XPS spectrum. The C *K*‐edge NEXAFS spectra nicely show the characteristic polarization dependence of the *π*‐ and *σ*‐resonances of graphene, with their line shape also suggesting out a weak coupling between copper and graphene. Figure 2f–h show the results of angle‐resolved photoelectron spectroscopy (ARPES) measurements performed on the same sample. These data reveal classical electronic landscape and graphene Dirac cone^[^ ^42^ ^]^ with a common electron doping shift. It is worth noting that a Shockley surface state can be clearly observed near the Fermi level (0.285 eV). This electronic state is another evidence of pure reduced copper, which surface is protected by a graphene layer. Such an observation is rather unusual for a CVD graphene on a copper catalyst. Note that this state, intrinsic to Cu (111) surface,^[^ ^43^ ^]^ has never been observed for as‐grown samples when non‐single‐crystal substrates were used.

Observation of an electronic surface state specific for (111) plane (Figure 2f) alone speaks in favor of a single‐crystal Cu formation. Underneath graphene, copper is clearly aligned, forming a single crystalline surface with [111] crystallographic orientation facing the sample's surface at 10–12° to the surface normal (as determined by Laue diffraction measurements, not shown). This observation is supported by the X‐ray diffraction (XRD, **Figure** **3**a–c) collected both in transmission (synchrotron radiation) and reflection (Mo Kɑ2 radiation) geometries. The observed angle between [111] direction and normal to the sample's surface can be understood by considering the interaction of carbon monoxide molecules with the specific cut of the catalyst's crystal lattice, at which CO adsorption energy is maximized.^[^ ^24^ ^]^ Such an unprecedented alignment facilitates the formation of single‐crystal grains of graphene, which is also confirmed by LEED obtained from individual graphene crystals of ≈4 mm in size (Figure 3d). The two hexagons in the diffraction pattern (rotated by roughly 7–8° with respect to each other) correspond to the copper (111) surface and a graphene grain over it with a typical mismatch.^[^ ^13^, ^44^, ^45^ ^]^ Electron backscatter diffraction (EBSD, Figure 3e,f) inverse pole figures shown in Figure 3e also reveal the high degree of orientation. Surprisingly, copper recrystallizes in this single‐crystal form, given that a polycrystalline molybdenum foil is used as a wetting layer. At the microscale, copper surface forms stepped structures as illustrated in Figure 3g captured by atomic force microscopy (AFM). The step size for the observed nanotiles is typically in the range of 1.5–3 nm. These steps also result in additional reflexes in the LEED pattern closer to the center of the image. Scanning tunneling microscopy (STM) captured at room temperature corresponds to the graphene lattice with *a* = 2.45 Å (Figure 3h) and reveals a Moire pattern caused by misalignment angle between (111) copper surface and graphene^[^ ^44^ ^]^ (Figure 3i). For more structural data see Figure S2 (Supporting Information). The peculiar interaction of carbon monoxide molecules with the catalyst's crystal structure not only facilitates the high degree of graphene crystallinity but also modifies the metal surface, making the graphene‐copper composite a promising material for catalysis. In general, the formation of a single graphitic layer may occur on other transition metals, such as Pd, Co, Fe, Pt (see Figure S3, Supporting Information). For most of them, the high adsorption energy‐driven interaction of carbon monoxide with the crystal structure has been reported.^[^ ^23^ ^]^ No D mode (present in Figure 1g) was observed in the Raman spectrum for all these materials. Most probably, defects, which are the reason for this band appearance, are caused by the transfer procedure.

A single crystal graphene synthesis is a result of a thorough kinetic study for the mechanisms of grain nucleation and growth. When working with copper foils, we have observed the graphene nucleation to be extremely sensitive to the surface roughness or impurities; we even observed the formation of the multilayer graphene (see Figure S4, Supporting Information). This is why we replaced the copper foil with a similar pure solidified^[^ ^14^, ^15^ ^]^ copper pellet on top of a molybdenum foil (acting as a wetting layer by heating to 1135 °C and cooling to 1085 °C) and added a procedure of CO_2_ annealing during the melting phase for additional copper cleaning. It should be stressed that we observed no traces of carbon species on Cu after CO_2_ annealing with XPS and Raman spectroscopy. Thereby, the rate of graphene nucleation was reduced by recrystallization of copper by several orders of magnitude reaching 10^2^–10^3^ cm^−2^s^−1^ while no impurities leading to multilayer graphene were observed as well.

**Figure 4:**
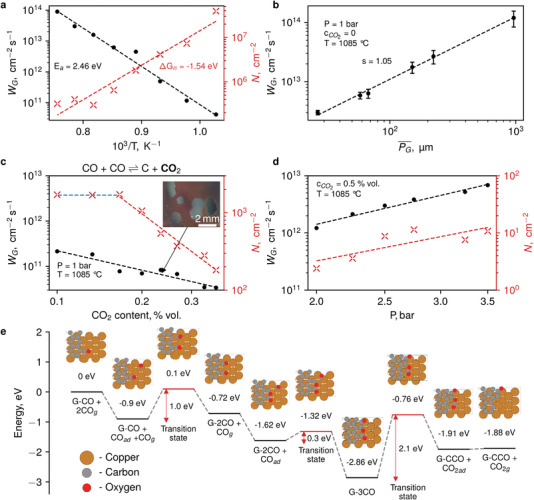
Synthesis kinetics toward graphene single crystals. a) Growth rate (solid black circles) and nuclei density (red crosses) dependencies on the synthesis temperature. Dashed lines indicate Arrhenius ln(*W*) ∝ 1/*T* fits with the corresponding activation energy and Gibbs free energy change indicated. b) The dependence of the growth rate on the average nucleus perimeter, indicating a self‐ripening growth regime. c) Reduction of the nuclei density and growth rate with increasing CO_2_ concentration due to the shift of equilibrium of the Boudouard reaction. The inset is a microphotograph of a sample grown in the low nucleation regime. d) The dependence of growth rate and nuclei density on the chamber pressure under the conditions of suppressed nucleation. e) Results of density functional theory (DFT) calculations illustrate the most probable route for a rate‐limiting reaction.

The temperature dependences of the growth rate (*W*_G_) and nuclei surface density (*N*) (**Figure** **4**a and Figure S5, Supporting Information) reveal the apparent activation energy *E*_a_ of 2.46 eV for the growth and observed change of the Gibbs free energy Δ*G* for the nucleation of −1.32 eV. Thus, higher temperatures favor the faster formation of larger crystals, though at *T* > 1085 °C (melting point for copper) graphene synthesis terminates. The dependence of the growth rate on the average grain perimeter (Figure 4b and Figure S5, Supporting Information) reveals a self‐acceleration of the growth together with an increase of the individual size of the grains. Moreover, it implies that a rate‐limiting step (if any) for the graphene synthesis happens on the grain border. According to the literature, the growth rate can be limited by several processes,^[^ ^46^ ^]^ including CO chemisorption (*E*_ad_ = 0.3–0.8 eV normally^[^ ^47^, ^48^ ^]^ and up to 1.8 eV in exotic cases^[^ ^49^ ^]^), diffusion^[^ ^50^ ^]^ (*E*_d_ = 1 eV), catalytic decomposition of CO molecule (*E*_dec_ = 2–4 eV),^[^ ^51^, ^52^ ^]^ and attachment^[^ ^53^ ^]^ of the carbon atom to the growing front of the nucleus (*E*_att_ = 2 eV). The last process is more likely to contribute the most to the limiting process.

To highlight the reaction mechanism, we have performed an investigation of CO dissociation on Cu (110) surface using density functional theory (DFT) methods (more details can be found in Figure S6, Supporting Information). We intentionally choose the (110) surface due to the following experimental facts. First, the graphene synthesis takes place at elevated temperatures, when the copper is melted and therefore can be presented as (110), the closest analogue to an amorphous surface. Second, we observed that solidified copper has (111) surface tilted relative to the perpendicular orientation of the sample, forming a complex stepped structure. This additionally motivated us to apply for our calculations the (110) surface. The barrier for CO surface decomposition was found to be 3.4 eV (or 3 eV on a defective Cu surface). On the contrary, CO dissociation at the graphene edge requires much lower energies; an adsorbed carbon monoxide relatively easily associates with a grain (1.0 eV for unsaturated surface, while only 0.3 eV for the complete saturation). Following CO_2_ removal shows the highest barrier in the sequence (2.1 eV for saturated surface (Figure 4e) and 2.3 eV in case of partial CO association with graphene (Figure S6, Supporting Information)). It should be noted that the calculations for (111) give merely the same barriers (more details can be found in the Supporting Information). This fact speaks in favor of the pivotal role of the graphene growth process when using CO as a carbon source. Nevertheless, neither utilization of (100), nor (111) can guarantee an acurate description—there is still probability of some additional processes occurring on the molten surface.^[^ ^54^ ^]^

Small amounts of CO_2_ are known to serve as an effective mild etchant affecting the carbon nanotube synthesis based on the Boudouard reaction.^[^ ^28^, ^29^ ^]^ As a nucleation process requires an oversaturation with intermediates, it is more sensitive to the concentration of the source species. Thus, the addition of less than 1 vol% of CO_2_ drops the graphene nuclei density by three orders of magnitude (Figure 4c) while the growth rate varies only by a factor of 2, which allows the synthesis of large crystals (several mm) simply by increasing the synthesis time up to ≈1.5 h. Nevertheless, running the synthesis at an elevated pressure^[^ ^13^ ^]^ (2–3 bar) allows to significantly reduce the time (down to 10 min) required for millimeter size crystals (Figure 4d). Thus, similar to the HiPco process^[^ ^30^ ^]^ for single‐walled carbon nanotubes (20–30 atm), a smooth balance between CO_2_ concentration and total pressure value is the key for facile synthesis of single‐crystal graphene.

**Figure 5:**
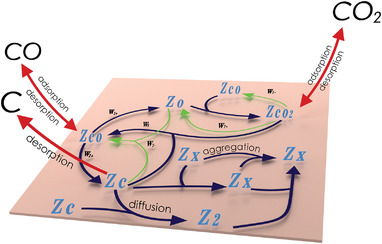
Scheme of the chemical reactions during the graphene nucleation (according to the considered model). Z_CO_, Z_CO2_, Z_C_, and Z_O_ correspond to adsorbed forms of the CO, CO_2_, C, and O correspondingly; Z_2_—adsorbed form of C_2_ dimer, while Z_x_—nuclei of graphene larger than two carbon atoms; surface reaction denoted with navy and green (some reverse processes for the sake of simplicity); while adsorption/desorption processes designated with red.

To reveal the graphene nucleation mechanism for the process based on the Boudouard reaction, we developed a model following Donohoe and Robins formalism.^[^ ^55^ ^]^ **Figure** **5** illustrates the processes considered within the model while the full description of formal kinetics as well as chemical equations can be found in the Supporting Information. Using a steady‐state approximation for carbon‐ and oxygen‐based intermediates, we derive the rate of nucleation (*dθ*_x_/*dt*):

(1) $$\begin{array}{ccc} \frac{d\theta_{x}}{dt} & = & \frac{k_{\mathrm{diff}}W_{2+}}{\left(k_{\mathrm{diff}}\theta_{x}+k_{\mathrm{des}}+\left(\frac{k_{2-}k_{7-}\theta }{k_{7+}\theta_{CO}}+k_{8}\right)\theta_{CO_{2}}\right)}\\ & & \left(\frac{W_{2+}}{\left(k_{\mathrm{diff}}\theta_{x}+k_{\mathrm{des}}+\left(\frac{k_{2-}k_{7-}\theta }{k_{7+}\theta_{\mathrm{CO}}}+k_{8}\right)\theta_{CO_{2}}\right)}-\beta \theta_{x}^{2}\right) \end{array}$$

where *θ*_x_, *θ*_CO_, $\theta_{CO_{2}}$, *θ*—surface coverages for carbon nuclei, CO, CO_2_, and free surface, respectively; W_2+_—the rate for decomposition of an adsorbed CO, *k*_diff_, *k*_des_—kinetic constants for diffusion and desorption of carbon intermediates; *β*—arbitrary constant for the diffusion of carbon intermediates and carbon dimers and nuclei, which can be estimated experimentally; *k*_7 +_ , *k*_7 −_ —kinetic constants for CO_2_ surface assembly from CO and O and decomposition, respectively, *k*_8_—kinetic constants for CO_2_ etching of carbon intermediates.

As for the experiments on nucleation temperature dependence, the carbon dioxide concentration was zero, we can omit corresponding terms to describe two cases denoted as “high” and “low” temperature by Donohoe and Robins.^[^ ^55^ ^]^

**Table:** 

*k*_diff_ *θ*_x_ ≪ *k*_des_(high *T*)	*k*_diff_ *θ*_x_ ≫ *k*_des_(low *T*)
$\frac{d\theta_{x}}{dt}\approx \beta k_{\mathrm{diff}}\frac{W_{2+}}{k_{\mathrm{des}}}\left(\frac{W_{2+}}{\beta k_{\mathrm{des}}}-\theta_{x}^{2}\right)$	$\frac{d\theta_{x}}{dt}\approx k_{\mathrm{diff}}W_{2+}\left(\frac{W_{2+}-\beta k_{\mathrm{diff}}\theta_{x}^{3}}{k_{\mathrm{diff}}^{2}\theta_{x}^{2}}\right)$
Solving the kinetic equation and assessing the effective Gibbs free energy for the nucleation (Δ_N_ *G*_eff_) via the temperature dependence of the nuclei density, we obtain the following combination of activation energies (*E*_a_) for the corresponding processes:	
$\lim_{t\to \infty }\theta_{x}=\sqrt{\frac{W_{2+}}{\beta k_{\mathrm{des}}}}$ $\Delta_{N}G_{\mathrm{eff}}=\;RT^{2}\;\frac{d\ln\theta_{x}}{dT}=\frac{E_{a_{2+}}-E_{a_{\mathrm{des}}}}{2}$ Based on data in ^[^^53]^ $E_{a_{\mathrm{des}}}=\;6\;\mathrm{eV}$ and DFT estimated barrier for CO cleavage ($E_{a_{2+}}$ = 3.3 eV) Δ_N_ *G*_eff_(high *T*) ≈ −1.35 eV ≤ 0	$\lim_{t\to \infty }\theta_{x}=\sqrt[{3}]{\frac{W_{2+}}{\beta k_{\mathrm{diff}}}}$ $\Delta_{N}G_{\mathrm{eff}}=\;RT^{2}\;\frac{d\ln\theta_{x}}{dT}=\frac{E_{a_{2+}}-E_{a_{\mathrm{diff}}}}{3}$ Based on DFT calculations, the barrier for CO cleavage $E_{a_{2+}}$ is of 3.3 eV: $E_{a_{\mathrm{diff}}}=0.6÷1.4\;\mathrm{eV}$ or even smaller; Δ_N_ *G*_eff_(low *T*) ≈ 0.6 ÷0.9 eV > 0

Unlike methane‐based graphene synthesis,^[^ ^53^ ^]^ we did not observe in experiment two distinct regimes for nucleation, only “high temperature one.” We attribute this to the high stability of CO molecule that prevents decomposition at conditions where diffusion controls the nucleation. Moreover, depending on CO_2_ concentration, we observe two distinct regimes meeting the experimental findings: CO_2_‐independent (blue in Figure 4c; domination of the first term in the denominator) and CO_2_‐sensitive regimes (red in Figure 4c):

(2) $$\lim_{t\to \infty }\theta_{x}=\sqrt{\frac{W_{2+}}{\left(\beta k_{\mathrm{des}}+\left(\frac{k_{2-}k_{7-}\theta }{k_{7+}\theta_{\mathrm{CO}}}+k_{8}\right)\theta_{CO_{2}}\right)}}$$

Thus, we propose the graphene nucleation to be most likely defined by CO decomposition and desorption of carbon‐based intermediates, while the addition of carbon dioxide leads to CO_2_‐independent and CO_2_‐sensitive regimes.

Interestingly, exceeding the marginal value of CO_2_ concentration (0.35% vol.) does not lead to the complete termination of the growth, but rather to a self‐etching of individual crystals. Even more, the critical concentration of CO_2_ (when self‐etching starts, see Figure S7, Supporting Information) depends on the overall pressure and varies in the range of 0.35 (1 bar) to 0.6% vol. (3.5 bar). It is worth mentioning that this behavior can be used for patterning of graphene grains paving the way for applications in gas sensors, catalysis, etc.

**Figure 6:**
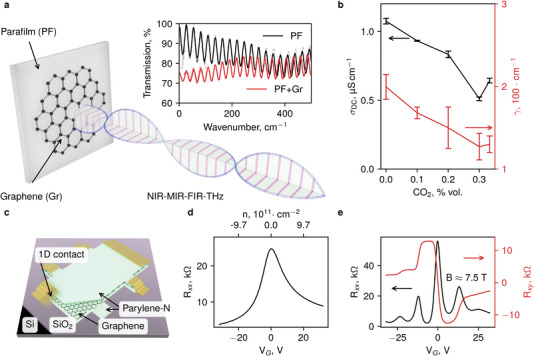
The electrical properties of transferred graphene. a) Schematic representation of the experiment. Graphene transferred to a PF substrate is subjected to electromagnetic radiation. The graph shows the typical transmission spectra of 134 µm‐thick PF and the same PF with transferred graphene (PF + Gr), shown in the region where absorption due to free electrons dominates. b) The dependence of the Drude model parameters (the static conductivity *σ*_DC_ and the scattering rate *ɣ*) of graphene synthesized at 1085 °C on CO_2_ concentration, obtained via THz spectroscopy. c) Schematic illustration of graphene devices with Au/Cr side‐contacts in a Hall‐bar geometry encapsulated in parylene‐N. d) A typical transconductance curve recorded at 300 K for 0.5 mm long and 0.3 mm wide channel. e) Hallmarks of the quantum Hall effect measured at 4.2 K for the 0.5 × 0.3mm hall bar structure of parylene‐N encapsulated graphene

Next, we investigated the charge carrier transport in the transferred graphene by means of contactless Fourier‐transform far‐infrared (FIR) and time‐domain terahertz (THz) spectroscopy and measurements of the field‐effect transistors (FETs) based on graphene with Au/Cr side‐contacts in a Hall‐bar geometry. In the latter devices, the graphene single crystal is encapsulated in parylene‐N, and the heavily doped Si substrate is used as a back gate. To obtain electrodynamic properties of synthesized graphene, we have measured transmission coefficient spectra of graphene supported by a parafilm (PF) substrate and that of a substrate alone (**Figure** **6**a and Figure S8, Supporting Information) after graphene was removed by oxygen plasma treatment (see Supporting Information). Oscillations below ≈600 cm^−1^ are due to the interference of the radiation within the plane‐parallel PF substrate (the Fabry–Perot effect). Above 500 cm^−1^ the spectra practically coincide, while the difference below ≈500 cm^−1^ is due to free charge carriers in the graphene layer (Figure S8, Supporting Information). Fitting these data with the Drude expression for conductivity has allowed us to extract parameters of graphene charge carriers. Drude conductivity is expressed as *σ*_AC_(ω) = *σ*_DC_ × [1 − *iωτ*]^−1^,^[^ ^56^ ^]^ where *σ*_DC_ = *neμ* = *ne* ^2^/*ɣm*_e_* is the direct current conductivity, *n* is charge carrier concentration, *ɣ* = 1/(2*πτ*) is charge carriers scattering frequency (inverse scattering time *τ*), *m*_e_* is the electron effective mass, and *μ* is the electron mobility. The calculated parameters of the Drude‐model fit for the samples grown with different CO_2_ concentrations are shown in Figure 6b. The scattering rate decreases with CO_2_ concentration (the red curve), as one would expect, since adding CO_2_ promotes the growth of greater graphene crystals. At the same time, the conductivity value decreases (the black curve), which suggests a decreasing charge carrier concentration. The obtained UV‐vis‐NIR spectra of the samples are consistent with the single‐layer nature of graphene: indeed, the *π* plasmon is located at ≈270 nm, and the transmittance is close to 97.7% (Figure S8, Supporting Information). In order to observe the hallmark features of graphene—an ambipolar transconductance characteristic and quantum Hall effect , we have fabricated 0.5 × 0.25 mm^2^ Hall‐bar structures from the individual graphene grains transferred from the catalyst and encapsulated in parylene‐N,^[^ ^57^ ^]^ which is one of the best encapsulation materials available for large‐size graphene samples (Figure 6c). These devices exhibit carrier mobilities of ≈2000 cm^2^V^−1^s^−1^ at ambient conditions and at low temperatures down to 0.5 K as obtained both from the FET measurements and the classic Hall effect. These results are summarized in Figure 6d,e. The relatively low mobility values might be associated with the damage of graphene during the transfer process (e.g., multiple wrinkles formed on the surface, as shown in Figure S9, Supporting Information), which is the problem not directly related to the synthesis procedure. Indeed, an analysis of THz‐FIR spectra following earlier reported protocols^[^ ^10^ ^]^ gives drift mobility values in the range of (1 ÷ 4) × 10^4^ cm^2^V^−1^s^−1^, likely indicating that the graphene transfer on PF affected its intrinsic properties. Subsequent transfer to Si/SiO_2_ substrate most likely caused more cracks and further decrease in the mobility. Clearly observable Dirac cone with *v*_f_ = 10^6^ m s^−1^ also indicates high quality of as‐grown graphene electronic properties.

From the engineering point of view, the utilization of CO simplifies the synthesis procedure: there is no need in vacuum^[^ ^8^, ^9^, ^10^, ^11^, ^12^ ^]^ or additional chemicals,^[^ ^8^ ^]^ and eventually, the number of tuning parameters (pressure, flow, gas composition, temperature) is reduced. Furthermore, the proposed procedure shows significant reduction in duration of heating and cooling procedures to a few tens of minutes. The catalyst preparation step, which might take 7–18 h,^[^ ^11^ ^]^ is reduced to 15 min. At the same time, high and competitive quality of the produced graphene can be easily achieved.

Future development of the proposed technique might include the design of one‐time‐fill operation under elevated pressure in the autoclave‐bomb of a cold‐wall reactor in order to reach extreme growth rates, which potentially can be upscaled to the wafer size graphene grown in a matter of several minutes. Aside from the well‐known applications of graphene in optoelectronic devices (assuming optimized transfer protocol), the reported novel synthesis opens several new avenues for the scientific community to explore. To name a few, the self‐etching mechanism of Boudouard reaction equilibrium shifting by CO_2_ allows synthesis of patterned graphene full of holes with perfect boundaries.^[^ ^58^ ^]^ The presented approach also allows further investigation of transition metal's electronic surface states protected by the graphene layer from degradation. Specific chemistry of various elements with carbon monoxide may allow formation of transfer‐free graphene, e.g. Ti + 2CO ⇆ TiO_2_ + 2C,^[^ ^59^ ^]^ TiCl_4_ + 2CO ⇆ TiO_2_ + 2C + 2Cl_2_ or Al_2_S_3_ + 3CO ⇆ Al_2_O_3_ + 3C + 3S^[^ ^19^ ^]^ which has been demonstrated for aluminum sulfide powder and can be extended to an atomic layer deposition or CVD films of aluminum sulfide.

## 3 Conclusion

To sum up, we show carbon monoxide to be a promising carbon source for the naturally self‐limiting synthesis of graphene on metallic substrates with low carbon solubility. High temperature annealing of the Cu surface in CO facilitates both a high degree of crystallinity of the formed graphene and modifies the catalyst resulting in the single‐crystal formation. The experimental design and thorough engineering of the growth governed by the Boudouard reaction allowed us to dramatically inhibit the nucleation rate reaching the regime of single‐crystal graphene growth highlighting the route toward the high pressure CO process—HiPco‐G. Besides, it also significantly streamlines the reactor design—operation at ambient or elevated pressures, absence of hydrogen, tuning a smaller set of parameters (synthesis temperature, pressure, CO_2_ concentration) altogether make the system safe and easy‐to‐build when compared to the hydrocarbon‐derived routes.

## 4 Experimental Section

### Graphene Synthesis

Chemical vapor deposition hot‐wall reactor was implemented in the form of a horizontal tube furnace with corundum tubular chamber. One of the tube flanges is connected to the set of mass flow controllers to introduce high purity Ar, CO, N_2_, and CO_2_ gases into the chamber. The other water‐cooled flange is connected to a home‐built gas‐tight inlet of a loading block in the form of another corundum∖quartz tube. Gas‐tight inlet is capable of the enclosure when the loading block is completely inserted and facilitates the pressure increase up to 4 atm. The process is schematically illustrated in Figure 1. For graphene synthesis at low temperatures (below 1082 °C), Puratornic 99.999% pure copper foil (Alfa Aesar) was used. For resolidified copper substrates Molybdenum foil (99.9%, RusHim, Russia) was used as a wetting layer and copper pellets (99.999% pure, Kurt J. Lesker) placed on the foil. After placing the substrate into the quartz load‐in/out tube and hermitization of the reactor, the sample was purged with N_2_/Ar for 15 min and loaded to the hot zone of the reactor; the size of the sample was much smaller than the size of the isothermal zone of the oven. The substrate was heated to 1135 °C within 15 min in N_2_/Ar + CO_2_ (1000 and 100 sccm flow, respectively) to anneal, melt, and clean the surface. Subsequently, the furnace was cooled down to 1085 °C within 4 min and the atmosphere was changed to a combination of CO and CO_2_, depending on the desired sample type. Next, the sample was rapidly pulled out to a water‐cooled flange zone and kept for 10 min under the synthesis atmosphere (in the case of elevated‐pressure experiments, the pressure was reduced to the ambient at this stage). Finally, the chamber was purged with the inert gas and the sample was taken out for further analysis.

### Single Crystal Recipe

Ambient pressure: 1085 °C, 3000 sccm CO, 8 sccm CO_2_, during 3 h.

Elevated pressure: 1085 °C, 3000 sccm CO, 18 sccm CO_2_, during 12 min at 3500 mbar reactor pressure.

### Polycrystalline Monolayer Recipe

Ambient pressure: 1085 °C, 3000 sccm CO for 12 min.

Elevated pressure: 1085 °C, 1000 sccm CO, for less than 1 min at 3500 mbar reactor pressure.

### Sample Characterization

Hereinafter gas composition and synthesis time would be indicated for the samples produced at a fixed temperature of 1085 °C. The sample for Figure 1e,f was synthesized with 3000 sccm CO and 8 sccm CO_2_ during 3 h. The Raman spectra and mapping in Figures 1g and 2a were captured from another sample, synthesized by the same procedure and transferred to Si/SiO_2_ substrate. XPS spectra in Figure 2b were captured from the sample synthesized at 3000 sccm CO and 5 sccm CO_2_ within 1 h. NEXAFS and ARPES data in Figure 2c–h were obtained from the sample synthesized the same way. For XRD investigation shown in Figure 3a sample synthesized with 3000 sccm of CO and 3 sccm CO_2_ within 1 h was used, while for synchrotron studies in Figure 3b the sample was synthesized using pure CO at 3000 sccm within 30 min. For data shown in Figure 3c,f,g,h sample synthesized at 3000 sccm CO and 6 sccm CO_2_ for 2 h was used. For LEED measurements (Figure 3e) the sample was synthesized at 3000 sccm CO and 8 sccm CO_2_ during 3 h.

For the kinetics investigation, samples synthesized on the copper foil were used. For each temperature point on the graph (Figure 4a) foil was firstly annealed at 1050 °C, afterwards cooled down to the synthesis temperature and subjected to the pure CO atmosphere for a fixed time chosen, so that a significant amount of isolated graphene grains (more than 200) was observed on copper of 1 cm^2^. Samples were later analyzed by the means of AFM and SEM and images were processed by an ImageJ particle distribution analysis plugin. In order to track data shown in Figure 4b pure CO atmosphere was used with a resolidified copper substrate. Each point of the graph was averaged for at least 100 grains, except for the last one, where it was impossible to have unmerged grains. For Figure 4c at least 10 grains (more grains for lower CO_2_ concentration) per point were captured by SEM or optical microscope. For high pressure experiments the CO_2_ concentration was fixed at 0.5% vol and at least 20 grains were collected. These measurements were complicated for lower CO_2_ concentration by the fact of extremely high growth rates and inability to capture the time required for the synthesis regime when individual grains are observable.

### X‐Ray Diffraction

Diffraction experiments in reflection geometry have been performed at Institute of Solid State Physics of Russian Academy of Sciences (RAS) using monocrystal diffractometer Oxford Diffraction Gemini equipped by charge coupled device detector with resolution of 0.35 A. MoK*α* radiation with wavelength of 0.7136 A was used. Further X‐ray diffraction experiments were executed at beamlines No. 2 and No. 4^[^ ^60^ ^]^ of VEPP‐3 storage ring at Siberian Synchrotron and Terahertz Radiation Centre, Budker Institute of Nuclear Physics of Siberian Branch of RAS, Novosibirsk, Russian Federation. The beamline No. 2 is dedicated for powder diffraction experiments with high instrumental resolution and is equipped with Si(111) channel‐cut monochromator, sample holder moving along theta angle, and Ge(111) crystal‐analyzer mounted in front of scintillation detector on the 2*θ* arm of the diffractometer. The working wavelength is 0.154 nm. The beamline No. 4 operates with a wavelength of 0.03685 nm and the diffracted radiation is registered by 2D image plate detector MAR345. The sample is set in transmission mode and can be rotated around the axis perpendicular to the incident beam.

The experiment at beamline No. 2 was carried out in different modes including conventional *θ*–2*θ* scanning, 2*θ* scanning at fixed theta angle of 4°, *θ* scanning at fixed 2*θ* positions corresponding to Cu(111) and Cu(200) reflections, scanning with sample spinned around normal to the surface. The diffraction patterns of the sample were obtained at beamline No. 4 both in conventional transmission mode with no sample movement and with the sample rotated around the horizontal axis within the range of 30°.

### X‐Ray Photoelectron Spectroscopy

XPS spectra were collected by XPS spectrometer Kratos Axis Ultra DLD with spherical sector analyser, ion gun, ultraviolet and x‐ray sources. Experiments were conducted under ultra‐high vacuum 5 × 10^−10^–3 × 10^−9^ Torr using the irradiation of AlK*α* (mono) 1486.69 eV (energy resolution 0.48 eV, binding energy calibration on Ag 3d_5/2_ line). Binding energy of Cu 2p_3/2_ at 932.7 eV is typical of metallic copper, while spin‐orbit splitting with Δ = 19.7 eV, minor satellite features at ≈943 eV, and finally the position of Auger line Cu_LMM_ at kinetic energy of 918.7 eV limit the possibility of any chemical modification of the substrate during synthesis.

### Raman Spectroscopy

Raman spectroscopy was performed with the DXRxi Raman Imaging Microscope equipped with ×50 objective and a 532 nm excitation laser operating at 1 mW output power. Raman spectra were measured at several points along with the sample with the exposure time

### Low Energy Electron Diffraction

LEED embodied in Kratos Ultra DLD was used for tracking the crystallinity of the samples. The distortion of the image, clearly seen from the imperfection of the hexagons formed in the diffraction pattern of the graphene, is caused by the form of the lens, non‐flat samples’ surface and edge effects of the signal capturing camera.

### ARPES

ARPES measurements were carried out using linearly polarized undulator radiation at the U112‐2‐PGM beamline of BESSY‐II in Berlin. Photoelectrons were detected with a Scienta R8000 analyzer at the “One square” ARPES instrument, and the base pressure of the experimental setup was better than 1 × 10^−10^ mbar. The angular and energy resolutions of the photoemission experiments were 0.1° and 10 meV, respectively. The sample was introduced to the ultra‐high vacuum UHV chamber from the atmosphere, and before measurements, it was annealed at 300 °C for 20 min. During the measurements, the sample was kept at 20 K.

### NEXAFS

Room temperature NEXAFS measurements were performed at Russian‐German dipole beamline of BESSY‐II electron storage ring operated by Helmholtz–Zentrum Berlin für Materialien und Energie.^[^ ^61^ ^]^ NEXAFS spectra were recorded in total electron yield mode. The measurements were performed for the as‐loaded as well as for the annealed (≈300 °C) samples of graphene on copper.

### Broadband Optical Spectroscopy

UV‐vis spectrometer Perkin Elmer Lambda 1050 was used for visible and UV range transmittance spectra capture. The Bruker Vertex 80V was used for obtaining NIR‐MIR‐FIR transmissivity spectra. Room temperature measurements of THz spectra and far infrared spectra were performed on a Teraview TPS Spectra 3000 time‐domain THz spectrometer and Bruker Vertex V80 FTIR spectrometer, respectively. To aware impurity sorption from the atmosphere the measurements were carried out inside CryoMech commercial cold‐finger cryostat with Mylar windows after 1 day vacuumization at lower than 5 × 10^−6^ mbar pressure. Spectroscopic data were collected with Opus and TPS Spectra software and processed using least‐square fitting procedure with the home‐made WASF software. Deep minima at higher frequencies correspond to absorption lines of PF . The spectrum of the PF was processed using a well‐known expression for transmissivity of the plane‐parallel layer^[^ ^62^ ^]^ with the absorption minima modeled with Lorentzians. The obtained electrodynamic parameters of PF were employed for least‐square fitting of the transmission coefficient of graphene on PF using corresponding expression for two‐layered systems (fitting results are shown by solid lines in Figure S8, Supporting Information).^[^ ^62^ ^]^ The decrease toward low frequencies of the oscillating transmission coefficient signifies metal‐like character of graphene conductivity: lower transparency of the spectrum at low frequencies corresponds to larger conductivity of the film—typical feature in the frequency dependence of AC response of metals/conductors.

### Microscopy

Bruker Multimode V8 atomic force microscope was used for graphene grains visualization on early stages employing the difference in adhesion force, young modulus, and sample deformation maps obtained in Quantitative NanoMechanical Mapping PeakForce tapping mode. Bruker RTESPA300 cantilevers with *k* = 40 N m^−1^ were used. The structure and distribution of graphene flakes were also investigated by SEM (FEI Versa 3D) under the conditions of secondary electrons detection mode (ETD detector) and 10 kV electron beam accelerating voltage. For larger grains and various catalyst substrates, Jeol JSM‐7001F at acceleration voltages from 10 to 30 kV, as well as optical microscope Leica with copper oxidation contrasting (annealing the copper substrates with graphene at 130 °C within 10 min) were used. Dual beam scanning electron microscope Helios G4 (Advanced Imaging Core Facility, Skoltech) was used to capture EBSD maps. Low‐temperature JT‐SPM SPECS was used for atomic reoslution imaging of graphene samples.

### Image Processing

FIJI software with a built‐in function was used for particle size distribution measurements. All images were converted to 16‐bit and the contrast was thresholded so that the background stays white, and the particles are black. After processing the obtained perimeters and areas of the particles were averaged. For processing of Raman, UV‐vis, IR, and XPS were processed using python lmfit, rampy, pandas, matplotlib, and numpy packages.

### Parylene Deposition

CVD parylene deposition was performed in a home‐built set‐up. A quartz tube was heated by two individual horizontal tube furnaces. One zone was kept at 175 °C, where the parylene‐N dimer was kept in a quartz boat. The second zone was heated to 700 °C. Passing through the tube, sublimed parylene dimer undergoes the pyrolysis in the hot zone and polymerization (at a rate of ≈20 nm/min) in the room‐temperature zone holding the samples. The room‐temperature zone can be isolated by a valve, allowing precise control of the final parylene thickness. The system is pumped by an Edwards scroll pump via a liquid nitrogen cold trap. Silicon wafers coated with the parylene were used as a substrate for graphene FETs and subsequent additional deposition of parylene on top of graphene for encapsulation.

### Graphene Transfer

Poly(methyl methacrylate) (PMMA), cellulose acetate (CA), and parafilm M were employed as sacrificial layers during the transfer. Due to large adhesion between graphene and copper substrate, most probably caused by van der Waals interactions, this work failed to tune the electrochemical delamination and SpEED peeling of the graphene. However, able to use a combination of electrochemical etching of the Molybdenum‐Copper substrate in “Hell”‐etchant (30 mL H_3_PO_4_, 18 mL HNO_3_, 10 mL CH_3_COOH, 65 mL H_2_O). After the etching was complete, the sample was transferred to pure water to remove the etchant residuals and scooped it with Si/SiO_2_ wafer in case of cellulose acetate or PMMA or the PF was taken out by tweezers with graphene for air drying. Following removal of the sacrificial layer was performed in cold acetone for PMMA and CA within 12 h. For the case of PF, the polymer film with graphene was applied to the surface of substrate under mild pressure by a rubber roller and heated to 120 °C on a hotplate to ensure uniform coverage. Subsequently, the substrate was placed in o‐xylene and kept overnight at room temperature, transferred to cyclohexane at 70 °C for 2 more hours and again to o‐xylene at 120° for 2 h. Afterwards, the sample was rinsed by isopropanol, deionized water, and vacuum dried.

### Contact Measurements

Under ambient conditions transfer curves have been captured with the help of a probe station and Keysight B1500A Semiconductor Analyser. Low temperature measurements were carried out in a ^4^He cryostat (4.2 K) where a sample was in the vapor of ^3^He (exchange gas). Before cooling down an insert with the sample was pumped during >5 h with residual pressure <5 × 10^−4^ mbar. Either standard four‐probe low‐frequency lock‐in or DC techniques were used for electrical transport measurements.

### Density Functional Theory

DFT calculations were performed to assess the reaction pathway for the graphene synthesis based on Boudouard reaction on a copper surface. A periodic 3 × 3 surface unit cell for Cu surface was used and the Cu slab was 7 layers thick. All the calculations were done with the grid‐based projector‐augmented wave (GPAW) code^[^ ^63^, ^64^ ^]^ with the Perdew‐Burke‐Ernzerhof (PBE) approximation for the Exchange and Correlation energy.^[^ ^65^ ^]^ For most calculations only one k‐point was used; nevertheless, some results were tested with (2 × 2 × 1) k‐points and the relative energies changed only around 0.1 eV verifying the proposed procedure. Climbing Image Nudged Elastic Band (CI‐NEB) methods were implemented in atomic simulation environment module in GPAW.^[^ ^66^, ^67^ ^]^ All NEB calculations need the initial and final geometries while an estimation of the transition state (TS) is also beneficial. Linear initial trajectory interpolation usually with initial, TS, and final geometries was used. In some cases, the reacting molecules moved significantly and the reaction path could have two barriers, the NEB calculations were restarted so that it would contain only one transition state. As the process occurs at the temperatures slightly above the melting point for copper, the surface is likely liquefied.^[^ ^68^ ^]^ Since the accuracy of DFT models for the liquid metal surface is substantially lower than that for the solid structure, for the DFT studies Cu(110) surface was employed. Even though the exact values for the activation energies might slightly differ for the different surfaces, the main trend would remain the same.

## Conflict of Interest

The authors declare no conflict of interest.

## Supporting information

Supporting Information

Supporting Information

Supporting Information

## Data Availability

The data that support the findings of this study are available from the corresponding author upon reasonable request.
