# Supplementary material for: High‐Quality Graphene Using Boudouard Reaction
Source: Adv Sci (Weinh). 2022 Feb 20;9(12):2200217. doi: 10.1002/advs.202200217 (PMC9036046; doi:10.1002/advs.202200217)
Supplement: Supplementary file 1 — Supporting Information [file ADVS-9-2200217-s002.pdf]

Supporting Information

High-quality graphene using Boudouard Reaction

*Artem K. Grebenko, Dmitry V. Krasnikov, Anton V. Bubis, Vasily S. Stolyarov, Denis V. Vyalikh, Anna A. Makarova, Alexander Fedorov, Aisuluu Aitkulova, Alena A. Alekseeva, Evgeniia Gilshtein, Zakhar Bedran, Alexander N. Shmakov, Liudmila Alyabyeva, Rais N. Mozhchil, Andrey M. Ionov, Boris P. Gorshunov, Kari Laasonen, Vitaly Podzorov, Albert G. Nasibulin**

Supplementary Note 1 - DFT calculations

We estimated a number of pathways for the graphene reaction. The pathway with lowest barriers is shown in **Figure S6e**. Nevertheless, we wish to share here some other results that might be useful for the community to assess other pathways and for further studies.

CO dissociation

We first tested the CO disproportionation reaction 2CO(s) -> C(s) + CO_2(g). The two CO molecules were placed on the upper row on bridge sites. In the final state CO_2 was not bound to the surface and the realized C atom was on a bridge site. The barrier of this reaction was 3.4 eV. The transition state is quite complex including a very short C-C distance of 1.44 Å.

The disproportionation reaction can also happen as a first-order reaction to form adsorbed C atom and O intermediates. The Cu(110) surface contains Cu rows shown in **Figure S6a**. The CO molecule prefers to be on a bridge site on the upper row, with binding energy of 0.95 eV. The C and O atoms prefer high coordination sites on the lower rows. The lowest barrier we found was 3.3 eV.

Since the synthesis occurs at high temperatures, there can be defects on the Cu surface. We removed one Cu atom near the CO molecule. The reaction trajectory is similar as before but the barrier is somewhat lower, namely 3.0 eV and it is the lowest CO first-order dissociation barrier we found. The graphene can also grow from carbon of dissociated CO elsewhere on the Cu surface as it has barrier of 0.8 eV only. Nevertheless, high barriers for CO first-order decomposition imply that process to affect the nucleation step only.

To assess the inhibition of the second layer formation, we studied CO dissociation on the graphene surface. We only compute the reaction heat since it is very large. Even a single layer graphene on the copper enhances the reaction heat to 7.8 eV (on Cu the energy is 6.0 eV). Clearly the underlying Cu will stabilize the C and O binding, inhibiting CO dissociation on graphene surface. It should be mentioned that the dissociation of the graphene bonded CO shows much lower barriers: the CO dissociation reaction heat is 0.5 eV and the reaction barrier is 1.6 eV (**Figure 4e**). Thus, defects and impurities (that will induce defects due to curvature) might facilitate further CO decomposition that agrees with experimental data.

Graphene growth

As the activation barriers for CO dissociation on Cu are rather high, we studied association of CO molecules. For this, a narrow 1D periodic ribbon (width of one carbon hexagon) of graphene can be placed on top of the Cu 3x3 unit cell. Along the Cu row direction three hexagon rings can be fitted conveniently. The carbon ribbon has dangling bonds and CO to passivate them.

The reaction energy (heat) of CO to move from a bridge site on the Cu row to the ribbon (i.e. associate with graphene) is -0.06 eV and the reaction barrier is 1.03 eV. The final state of this system is shown in **Figure S6c** (and **Figure 4e** in the main text). For example, in the fully H passivated system the reaction energy is larger, 1.17 eV and the barrier was 1.51 eV. Interestingly this barrier was not much higher than in the case of the non-passivated site.

Low barriers for CO association raise the question on O removal (in form of atom or CO_2). First, we consider the CO dissociation to C and O associated with graphene edge. We found a low dissociation barrier (1.6 eV) of CO dissociation next to graphene but the oxygen removal is more difficult with a barrier of 2.6 eV (reaction energy of 0.44 eV). Another possibility is that two oxygen atoms close to each other on the graphene edge, graph-CO + graph-CO (or graph-O), can react and form an oxygen molecule. We did not perform NEB calculation for this reaction since the reaction energy of the reaction graph-CO + graph-O -> graph-C + graph + O_2 is very high, around 6 eV. This suggests that such a reaction path is not possible.

We consider another oxygen removal reaction with association of additional CO. O removal was proposed with gas phase CO (Eley-Readil like mechanism)[1]. The barrier of graph-O reaction with CO(g) was 2.1 eV and the reaction energy is close to 0 eV. The collision of CO from the gas is from the entropy point of view challenging. The molecule needs to come in precise angle to a relatively small area near the O. We did not observe in the NEB trajectory any attractive long-range attraction between the CO and graph-O. This should be compared to the CO binding to Cu surface where CO binds with very high probability. The surface bound CO's will diffuse with a low barrier (around 0.5 eV) near to the graphene. Even the O removal with CO(g) and CO(s) have similar barriers; the pre-factor is much larger in the case of CO(s).

We observed the lowest barriers for neighboring associated CO groups. From the $Graph(CO)_3$ model we investigated the removal of CO_2 from the ring structure. The barrier of this reaction is only 2.1 eV and the reaction energy is 0.95 eV **(Figure 4e).** There are several other possibilities to add CO to the graphite edge. **Figure S6g** shows the configuration, where graph-C and graph-CO formed a 5-ring with O bound to it. This system can further react with a CO molecule on Cu with the barrier of 2.2 eV and the reaction energy of 0.7 eV. We investigated several pathways to remove O with reaction barriers of 2.1 to 2.3 eV. The lowest barrier was found when all the edge carbons were bonded with CO and where one CO molecule and O atom from another CO formed a CO_2 molecule. As several O removal processes have close barriers, we assume that likely all these reactions might happen in the real growth process.

Some movies of the NEB calculations

To illustrate the NEB calculations, we made two movies.

One movie is to illustrate graph–O + CO -> graph + CO_2 reaction and another one for the graph–CO + CO -> graph-C + CO_2 reaction. The NEB energy profiles are in **Figure S6h** and **S6i**.

(Movie files: graph-O-CO-to-CO2.mp4 and graph-CO-CO-to-CO2.mp4)

Cu (111) surface

We made a model for graphene that contain 3 rings on Cu (111) surface illustrated in **Figure S6j**. We added the graphene model and a CO molecule to the Cu(111) surface and a performed a NEB calculation of the CO to attach to the site in graphene. The barrier of this reaction is only 0.34 eV and the reaction energy is -0.87 eV in the case of the highest site. There are also two other CO binding sites and the reaction energies to these are -0.20 eV and -0.40 eV. We also perform a NEB calculation to the third site. The barrier was 0.60 eV. These barriers are low and all the reaction energies are negative so it is very likely that some of the real graphene edge sites have CO bound to them.

Next, we investigated the CO_2 formation from a system where two COs are bound to the graphene model. The lowest CO_2 production reaction has barrier of 2.45 eV. The direct CO_2 production barriers are significantly lower than the disproportion reaction barrier on (111) surface. They are also similar to the barriers on the (110) surface. We can conclude that also in the (111) the graphene acts as a catalyst to its growth. Naturally the search of reaction in the (111) case is limited compared on the (110) surface but the (111) results support the original hypothesis.

Supplementary Note 2 – Nucleaction model

We developed a phenomenological kinetic nucleation model for graphene formation; it is heavily based on a classical publication by Donohoe and Robins[2]. It should be noted that though Kim et al.[3] use the Donohoe and Robins model as well, we extensively modified the equations for the Boudouard reaction maintaining the formalism of surface kinetics. Thus, the model rather corresponds to Donohoe and Robins[2] and even follows some of the assumptions.

Let us consider the graphene nucleation to proceed on an ideal (no effect of any defects or borders) surface of copper and surrounding atmosphere of CO and CO_2 (**Figure 5**). Except for intuitive reversible adsorption processes for CO and CO_2, the copper surface can host the decomposition of CO (to form adsorbed species of C and O) and CO_2 (to form adsorbed CO and O) as well as migration of adsorbed species and agglomeration of carbon atoms into dimers or larger C-based species. The role of CO_2 concentration in the present model is to control the surface concentration of carbon species via direct etching of carbon or indirect process by formation of oxygen intermediates. Following the formalism developed in Donohoe and Robins[3], we will consider C_2 species as stable nuclei (i.e. larger than the critical radius for nuclei). This allows us to neglect the possible cleavage of C-C bonds on the surface. The only counterintuitive reaction we consider to take place on the surface is C desorption (following Donohoe and Robins formalism as well).

The processes can be illustrated with a graphical scheme **(Figure 5)** and with the following equations.

1) $CO + Z \rightleftarrows Z_{CO} (CO\ adsorption)$

2) $Z_{CO} + Z \underset{k_{2-}}{\overset{k_{2+}}{\rightleftarrows}} Z_C + Z_O (CO\ decomposition)$

3) $Z_C + Z_C \xrightarrow{k_{diff}} Z_2$ $(nuclei\ formation; W_3)$
4) $Z_C + Z_x \xrightarrow{k_{diff}} Z_x$ $(nucleus\ elnargement; W_x)$
5) $Z_x + Z_x \xrightarrow{\beta k_{diff}} Z_x$ $(nucleus\ aggregation; W_{agg})$
6) $Z_C \xrightarrow{k_{des}} Z + C_{gas}$ $(desorbtion\ of\ C; W_{des})$
7) $Z_O + Z_{CO} \rightleftarrows Z_{CO_2} + Z$ $(CO_2\ formation)$
8) $Z_{CO_2} + Z_C \xrightarrow{k_8} 2Z_{CO}$ *(CO2 etching of carbon intermediates)*
9) $CO_2 + Z \rightleftarrows Z_{CO_2}$ $(CO_2\ adsorption)$

To retrieve the concentration of graphene nuclei, we need to estimate the surface coverage of carbon atoms (θ_C)[1]. Donohoe and Robins consider the coalescence of nuclei to be a superposition of Ostwald ripening and agglomeration. That is why the kinetic constant was set to be proportional to the diffusional constant of a single carbon atom (k_{diff}) surface coverage of carbon monomers Z_C and an arbitrary constant β which can be estimated experimentally.

$$\frac{d\theta_x}{dt} = W_3 - W_{agg} = k_{diff}\theta_C^2 - \beta k_{diff}\theta_C\theta_x^2 = k_{diff}\theta_C(\theta_C - \beta\theta_x^2)$$

As the adsorbed carbon and oxygen atoms are extremely labile, one can reasonably employ the steady-state approximations[1]:

$$\frac{d\theta_C}{dt} = 0;\ \frac{d\theta_O}{dt} = 0$$

As CO is a stable molecule, the surface coverage of labile carbon atoms can be reasonably considered to be significantly smaller $\theta_{CO} \gg \theta_C$ (this is also one of the requirements of the steady-state approximation as adsorbed CO is a source for adsorbed C[1]). As thek_{2-} and k_{7+} represent diffusive processes, they are likely to depend mostly on activation barriers defining diffusion coefficients: $k'_{diff} = f(D_O; D_{CO})\ and\ k_{2-} = f(D_O; D_{CO})$. DFT simulations show the CO and O to be weakly adsorbed compared to C leading us to the conclusion that $k'_{diff}\theta_{CO} \gg k_{2-\theta_C}$.

	E_a for diffusion, eV	
	Along (110) row	Between rows (110)
CO	0.10	0.64
C	0.60	1.44
O	0.35	0.18

$$\theta_O = \frac{W_{2+} + W_{7-}}{k_{2-}\theta_C + k_{7+}\theta_{CO}} \approx \frac{W_{2+} + W_{7-}}{k_{7+}\theta_{CO}}$$

According to Donohoe and Robins[7], the formation of dimers happens with a much lower probability than that desorption and attachment to larger nuclei:

$$\frac{d\theta_C}{dt} = 0 \rightarrow\ W_{2+} - W_{2-} = W_3 + W_x + W_{des} + W_8 \approx W_x + W_{des} + W_8$$

$$\theta_C = \frac{W_{2+}}{(k_{diff}\theta_x + k_{des} + \left(\frac{k_{2-}k_{7-}\theta}{k_{7+}\theta_{CO}} + k_8\right)\theta_{CO_2})}.$$

For the experiments on the temperature dependence of the nucleation the carbon dioxide concentration was zero. Thus, the final equation for the formation of the graphene nuclei is as follows

$$\frac{d\theta_x}{dt} = k_{diff}\frac{W_{2+}}{(k_{diff}\theta_x + k_{des})}\left(\frac{W_{2+}}{(k_{diff}\theta_x + k_{des})} - \beta\theta_x^2\right)$$

Two cases can be studied, which were shown "high temperature" and "low temperature" regimes in Donohoe and Robins[3] to yield into different observed concentrations of the nuclei ($\lim_{t\to\infty} \theta_x$):

$k_{diff}\theta_x \ll k_{des}(high\,T)$	$k_{diff}\theta_x \gg k_{des}(low\,T)$
$\frac{d\theta_x}{dt} \approx \beta k_{diff} \frac{W_{2+}}{k_{des}}\left(\frac{W_{2+}}{\beta k_{des}} - \theta_x^2\right)$	$\frac{d\theta_x}{dt} \approx k_{diff} W_{2+}\left(\frac{W_{2+} - \beta k_{diff}\theta_x^3}{k_{diff}^2\theta_x^2}\right)$

Solving the kinetic equation and assessing the effective Gibbs free energy for the nucleation ($\Delta_N G_{eff}$) via the temperature dependence of the nuclei density, we obtain the following combination of activation energies (E_a) for the corresponding processes:

High T:

$$\lim_{t\to\infty} \theta_x = \sqrt{\frac{W_{2+}}{\beta k_{des}}}$$

$$\Delta_N G_{eff} = RT^2 \frac{dln\theta_x}{dT} = \frac{E_{a_{2+}} - E_{a_{des}}}{2}$$

Based on data in [2] $E_{a_{des}} = 6\ eV$ and DFT estimated barrier for CO cleavage ($E_{a_{2+}}$ =3.3 eV).

$$\boldsymbol{\Delta_N G_{eff}(high\,T) \approx -1.35\ eV \leq 0}$$

Low T:

$$\lim_{t\to\infty} \theta_x = \sqrt[3]{\frac{W_{2+}}{\beta k_{diff}}}$$

$$\Delta_N G_{eff} = RT^2 \frac{dln\theta_x}{dT} = \frac{E_{a_{2+}} - E_{a_{diff}}}{3}$$

Based on DFT calculations, barrier for CO cleavage $E_{a_{2+}}$ is of 3.3 eV:$E_{a_{diff}} = 0.6 \div 1.4\ eV$ or even smaller.

$$\boldsymbol{\Delta_N G_{eff}(low\,T) \approx 0.6 \div 0.9\ eV > 0}$$

Thus, as the experimental value is of -1.54 eV (**Figure 4a**), most likely so-called high temperature regime takes place on the considered reaction. Moreover, if we consider the high temperature regime in the presence of CO_2, it allows to observe us a possibility of two distinctive zones for the nucleation: CO_2 independent (the first term (blue) dominates denominator) and CO_2 sensitive (the second term (red) dominates denominator). This agrees with the experimental data (**Figure 2c)** as two zones with (red line **Figure 2c**) and without CO_2 dependence (blue line **Figure 2c**) were observed.

$$\lim_{t\to\infty} \theta_x = \sqrt{\frac{W_{2+}}{\left(\beta k_{des} + \left(\frac{k_{2-}k_{7-}\theta}{k_{7+}\theta_{CO}} + k_8\right)\theta_{CO_2}\right)}}$$

Further analysis of, for example, CO and CO_2 concentration trends, is highly limited by a need for more precise estimations of all the kinetic constants, taking into account complex processes (e.g. direct oxidation carbon nuclei with carbon dioxide)[4,5] in the equation for high temperature approximation and could be an interesting topic for separate research.

Supplementary references

[1]. Kinetics In *Concepts Mod. Catal. Kinet.*, John Wiley & Sons, Ltd, **2003**, pp. 23–78.

[2]. A. J. Donohoe, J. L. Robins, *Thin Solid Films* **1976**, *33*, 363.

[3]. H. Kim, C. Mattevi, M. R. Calvo, J. C. Oberg, L. Artiglia, S. Agnoli, C. F. Hirjibehedin, M. Chhowalla, E. Saiz, *ACS Nano* **2012**, *6*, 3614.

[4]. V. Yu. Bychkov, Yu. P. Tyulenin, O. V. Krylov, V. N. Korchak, Kinet. Catal. 2002, 43, 724.

[5]. V. Yu. Bychkov, Yu. P. Tyulenin, V. N. Korchak, Kinet. Catal. 2003, 44, 353.

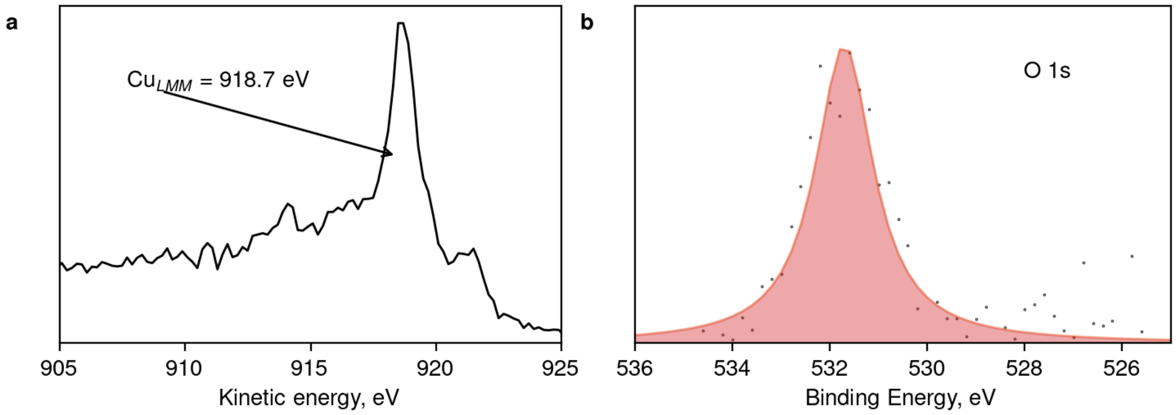

Figure S1. Additional XPS data. (**a**) Cu Auger line spectrum**.** Position of the Cu_{LMM} line speaks in favour of Cu^0 state of the catalyst underneath the graphene and supports findings indicated in **Figure 2**. (**b**) O 1s core level spectrum. According to later performed NEXAFS measurements this peak is assigned to atmospheric analytes.

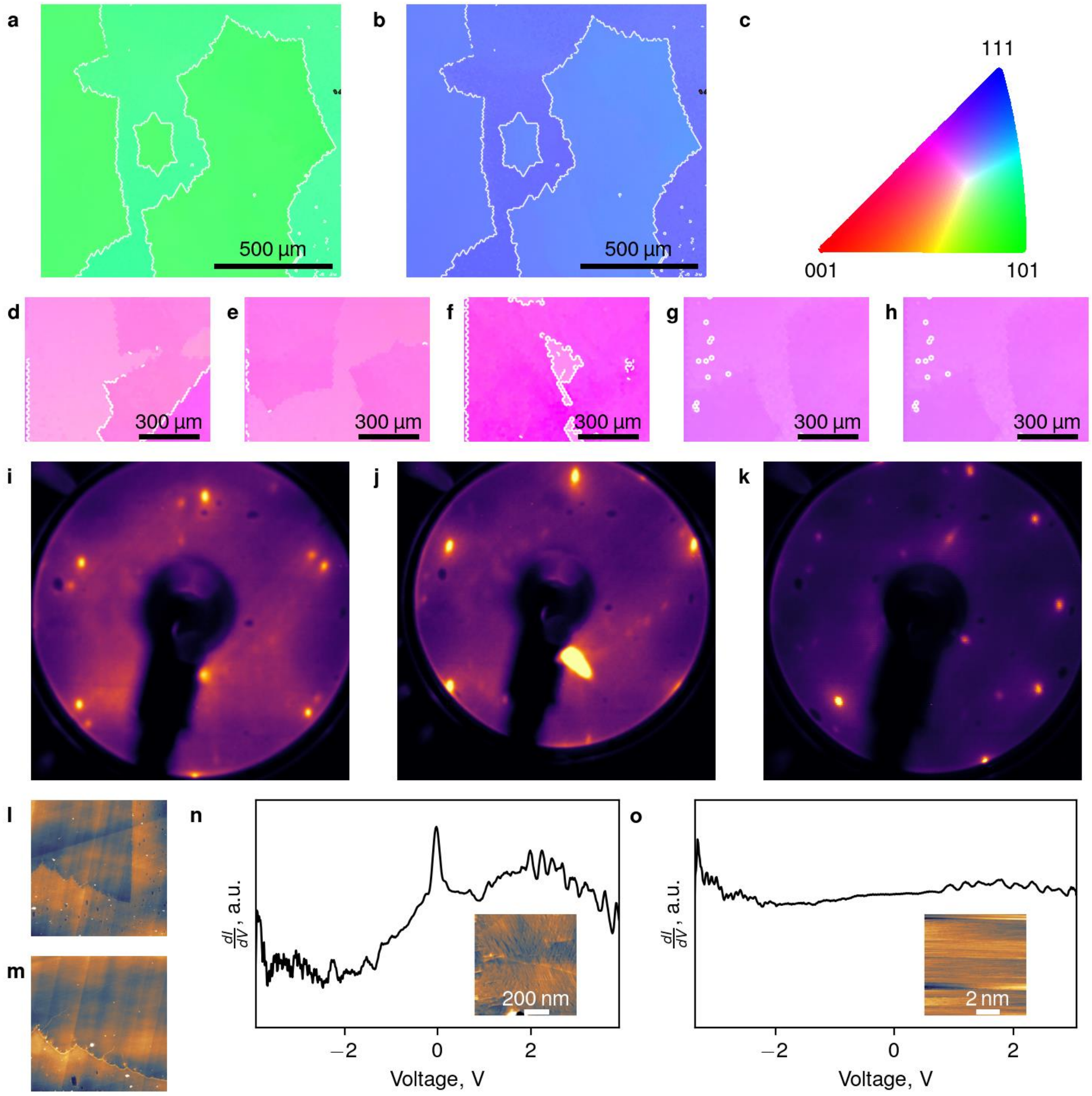

Figure S2. Additional structural data of Cu-graphene samples. (a-b) EBSD IPF maps for transverse and longitudinal directions respectively. (**d-h**) Additional EBSD IPF maps for normal direction. (**c**) indicates the color-legend. (**i**) and (**j**) LEED diffraction patterns of two more regions on the sample covered with individual graphene grains. (**k**) LEED pattern from the oxidized surface of Cu not covered by graphene. (**l**) and (**m**) Large scales (35 and 15 um fields respectively) of the same region where steps were captured. (**n**) and (**o**) LDOS curves from the region containing steps and flat region on the individual step respectively. Topography images are indicated in the insets of corresponding panels.

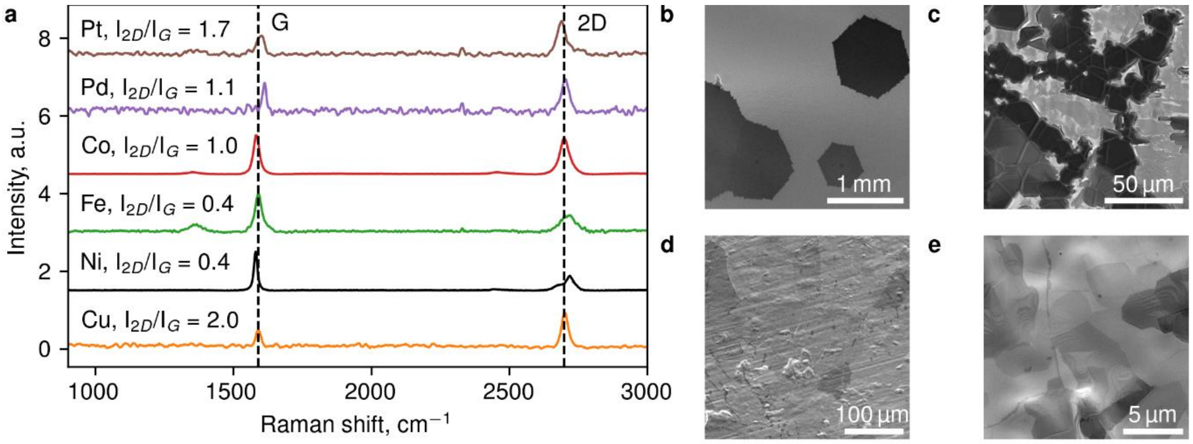

Figure S3. Catalytic decomposition of CO. (a) Raman spectroscopy of graphene-like structures obtained on various substrates. The background was removed. For most cases except the Cu, few-layered graphene is formed either due to the high solubility of carbon in these materials at elevated temperatures (Ni, Fe, Co) or due to highly developed surface (Pd, Pt) which caused the boost of nucleation on the impurities and protrusions. **(b-e)** SEM images of the graphene layers on the surface of copper (individual crystals), nickel, palladium and platinum respectively.

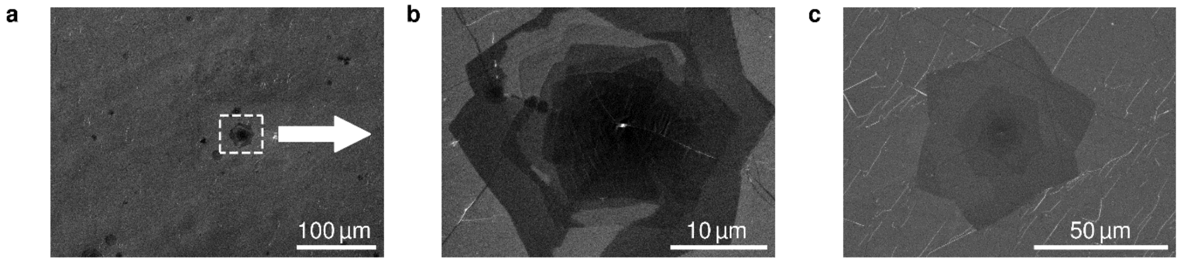

Figure S4. Influence of impurities and developed morphology on the multilayer graphene formation. (**a**)**,** SEM image of copper substrate covered with monolayer graphene. Growth time was intentionally chosen twice larger than required for complete coverage of the utilized substrate size (1x1 cm). (**b**) Close-up view of the selected region depicting multilayer graphene generation on the apex formed during solidification with extremely reduced growth rate. (**c**) Additional multilayer structure formed on the surface of the dirty catalyst.

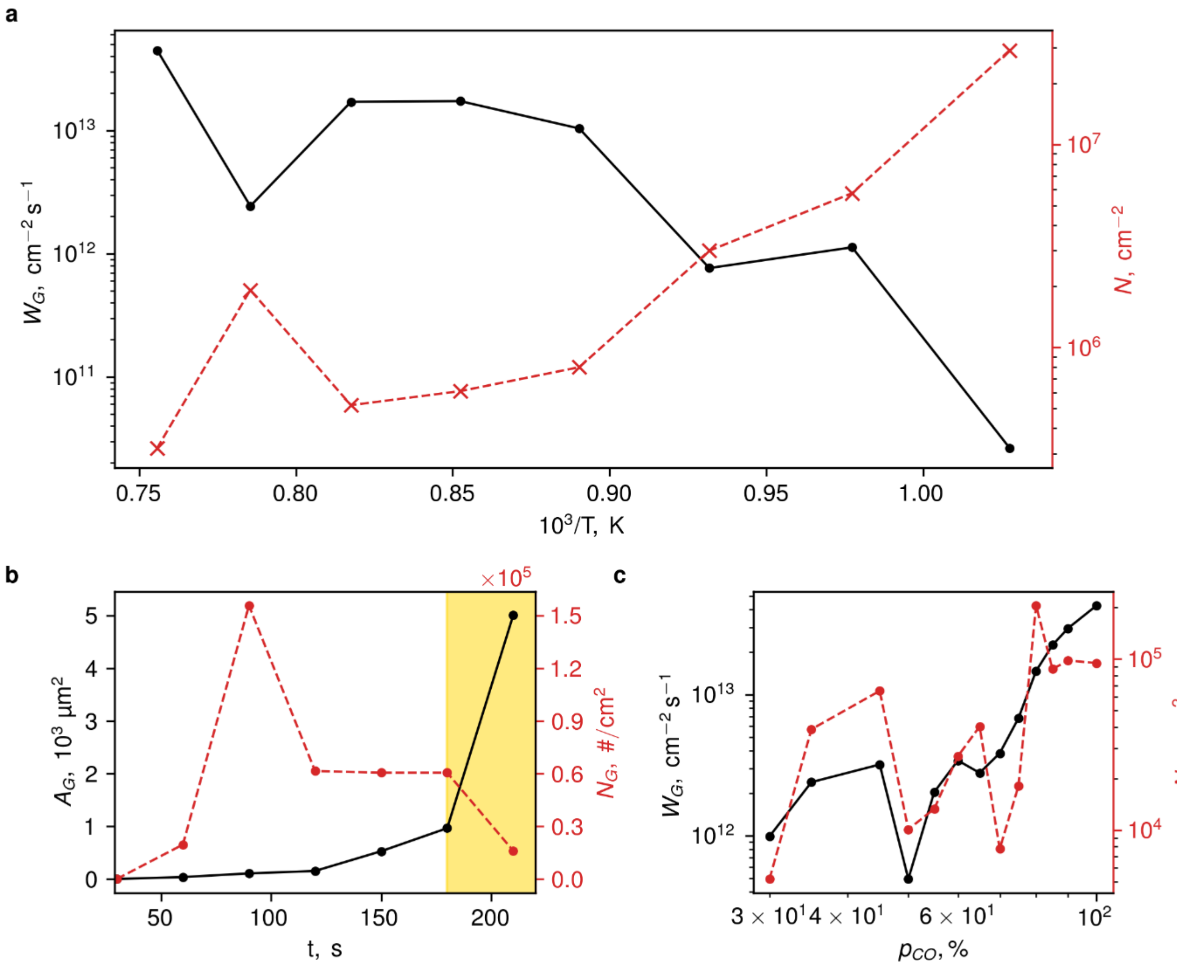

Figure S5. Additional information on the kinetics investigation. (a) Growth rate (W_G, $cm^{-2}s^{-1}$) and nuclei density (N, cm^{-2}) dependencies on the synthesis temperature for the situation when copper foil is heated only to the synthesis temperature. When compared to data shown in **Figure 4** it is clear that preconditioning removes non-monotonic behavior caused by the catalyst modification during annealing. (**b**) Dependence of the average area and number of nuclei on time. Yellow zone illustrates the region where individual grains start to merge. (**c**) The dependence of growth rate and nuclei density on the partial pressure of CO when the gas is diluted with Ar/N_2. Such an approach does not facilitate reaching the single-crystal regime.

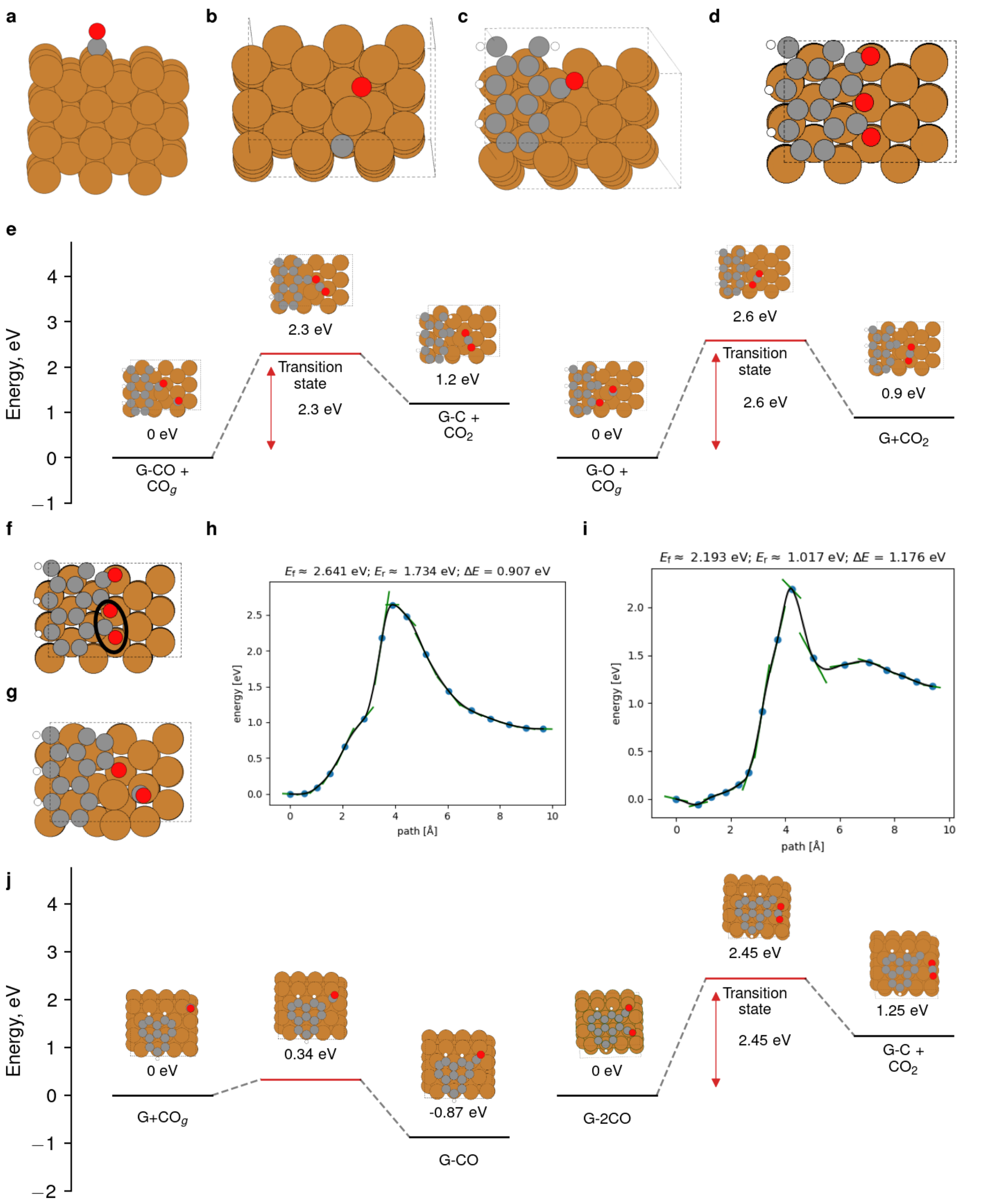

Figure S6. Additional Data for DFT calculations. (a), The model for Cu(110) surface with CO molecule on it. The brown spheres are the Cu atoms and grey and red spheres are the carbon and oxygen, respectively. (**b**), The final state of CO dissociation on Cu(110) surface. (**c**), A CO molecule bound to the graphene model GraphH. (**d**), The fully CO passivated graphene edge. Note that the CO's will form rings, (**e**), Oxygen removal reactions from graph-CO and graph-O. TS denotes the transition state. (**f**), The fully CO passivated graphene edge. The oval denotes the atoms to form the CO2 molecule. (**g**), Starting structure of the graph-C and graph-CO system is one where graph-C and graph-CO have formed a 5-ring with O bound to it. NEB energy profiles of the (**h**) graph–O + CO -> graph + CO_2 and (**i**) graph–CO + CO ->

graph + CO_2 reactions. (**j**) Energy diagrams for CO adsorption reaction and oxygen removal reaction for Cu (111).

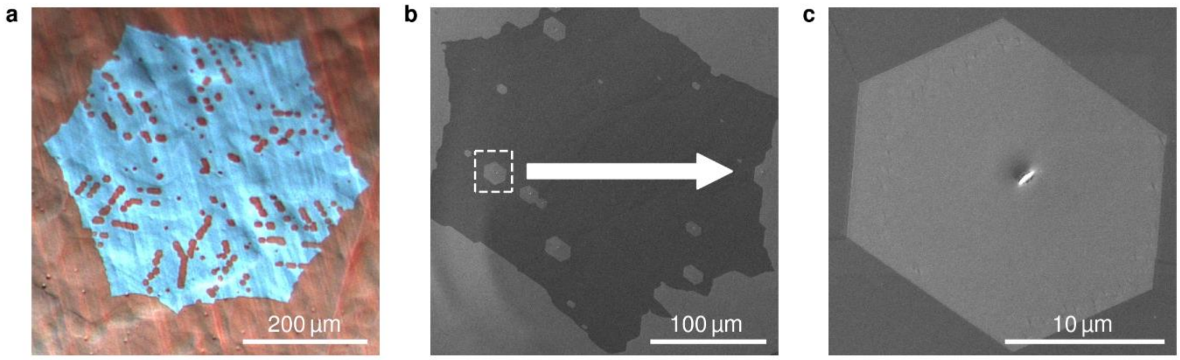

Figure S7. Self-etching of graphene crystals by carbon dioxide (c(CO_2)>0.35 % at P = 1 atm). (**a**) Optical microscopy image of individual graphene grain etched by carbon dioxide during growth. (**b**) SEM image of the analogous grain under the same processing conditions and (**c**) close-up on etched region with perfect boundaries.

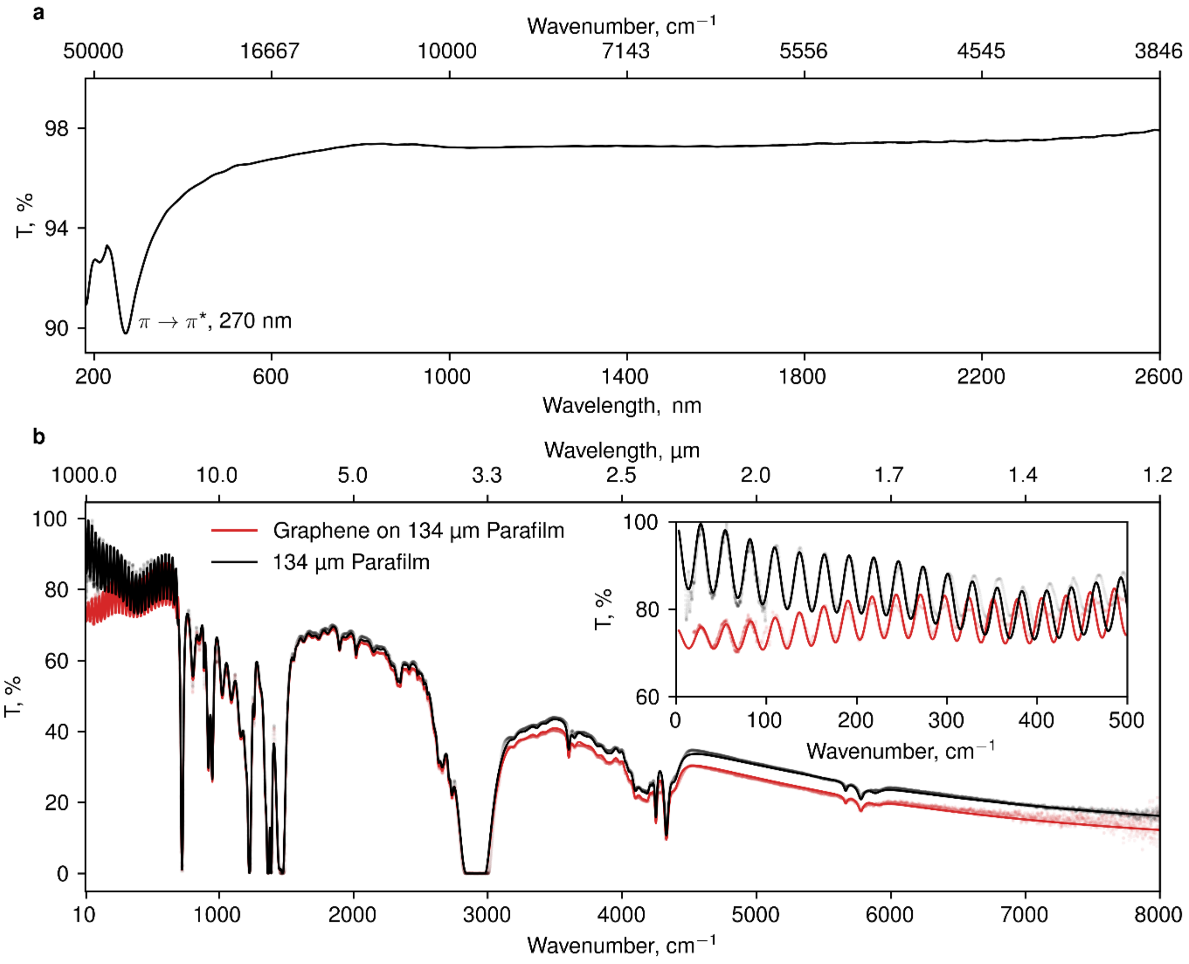

Figure S8. Broadband optical spectroscopy of transferred graphene. (a), UV-vis-NIR spectrum of the graphene sample transferred to the quartz substrate. (**b)**, NIR-MIR-FIR-THz transmission coefficient spectrum of the graphene on the 134 μm thick parafilm substrate. Deep minima above ≈500 cm^{-1} are due to molecular vibrations in parafilm substrate.In the inset, the region of interest is illustrated, where the presence of free charge carriers in graphene influences the transmissivity. Dots show experimental data, lines present least-square fit results.

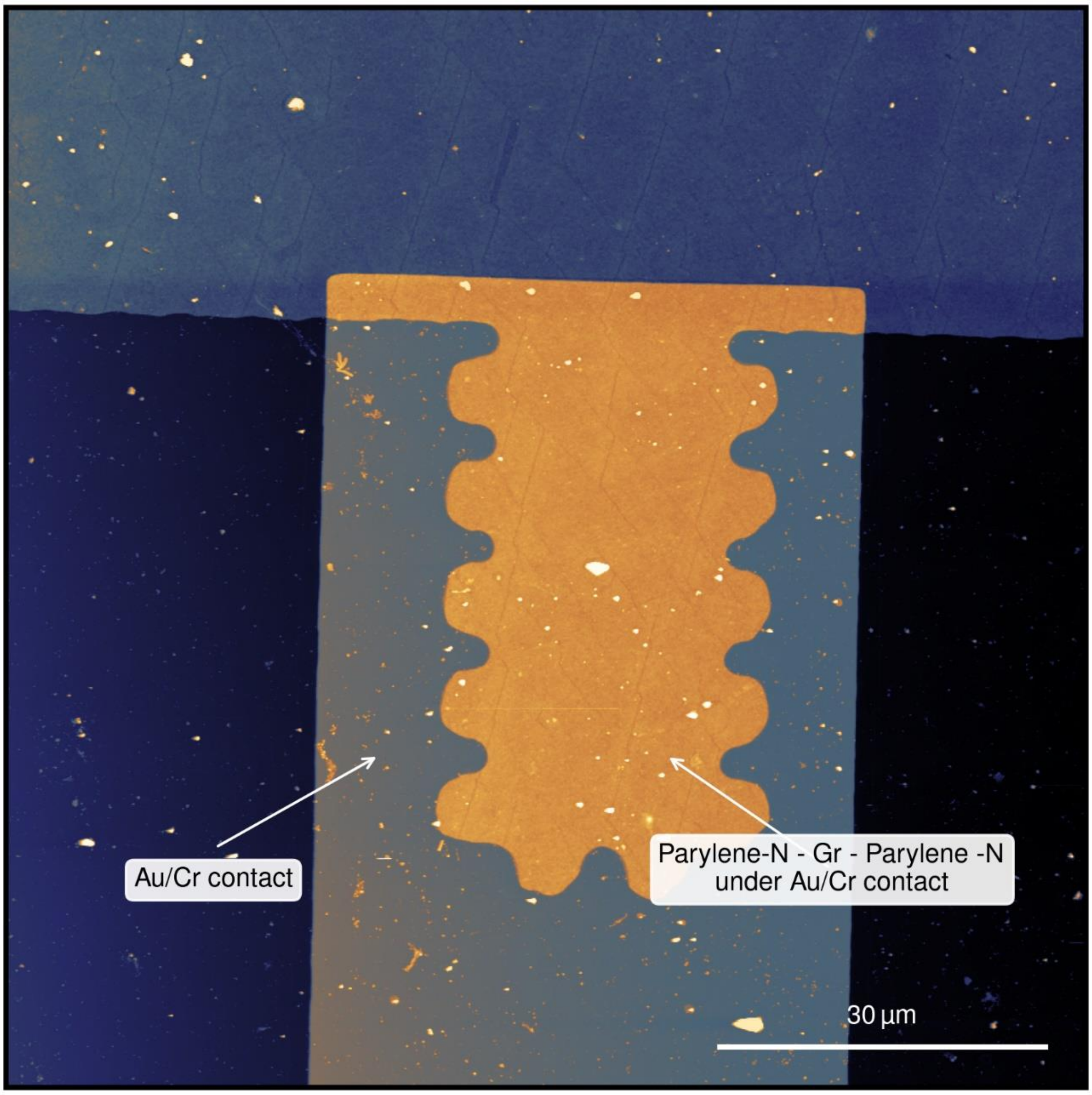

Figure S9. AFM analysis of parylene-N encapsulated graphene. Transfer procedure from the stepped surface of copper substrate results in multiple wrinkles and cracks clearly visible even through the encapsulation layers.

Movie S1.

A CO reacting with an oxygen atom bound to the graphene model. Barrier of this reaction is 0.91 eV.

Movie S2.

A CO reacting with a CO bound to the graphene model. Barrier of this reaction is 1.2 eV. In both cases the reacting CO is bound to the Cu surface.
